# Consumer behavior and insurer plan offering with expanded premium subsidy in U. S. individual health insurance market

**DOI:** 10.3389/fpubh.2025.1643109

**Published:** 2025-10-13

**Authors:** Yu Lei

**Affiliations:** Barney School of Business, University of Hartford, West Hartford, CT, United States

**Keywords:** health insurance enrollment, insurer plan offering, enhanced premium subsidy, metal tiers, consumer choice

## Abstract

As the demand for individual health insurance in the U. S. ebbed and flowed between 2014 and 2024, the supply of such coverage also fluctuated accordingly, based on our descriptive analysis of enrollment data and insurer information. With the enhanced premium subsidy available since 2021, consumers were trading up and purchasing more gold plans and less silver plans. Insurers in turn offered more of the desired plans. Our results also showed that insurers offered a higher percentage of silver plans but a lower percentage of bronze plans than the consumer demanded.

## Introduction

The 111th United States Congress enacted the Patient Protection and Affordable Care Act (also known as the Affordable Care Act, ACA) in March 2010 (1). This comprehensive healthcare reform law mandated the establishment of health insurance marketplaces in each state beginning in 2014 and eligible people may receive premium subsidy.

Marketplace plans have four metal categories (2), which cover the same set of essential benefits at different percentages. Bronze plans cover an average of 60% of the medical cost, silver 70%, gold 80%, and platinum 90%. There is also a catastrophic plan that is only available to people under 30 or people 30 or older with a hardship exemption or affordability exemption.

The premium subsidy (also known as tax credit) is the difference between the premium for a benchmark plan and the insured’s expected premium contribution. The second lowest silver plan is used as the benchmark plan to calculate premium tax credit, which can be used to purchase any metal plan, not just silver. The insured’s expected premium contribution is a certain percentage^1^ of household income.

From 2014 to 2020, only people with incomes under 400% of the federal poverty level (FPL) may be eligible for premium subsidy. After the American Rescue Plan Act (ARP) was enacted in 2021 (3), the premium subsidy was expanded to people making 400% of FPL or more and increased the subsidy amount to existing eligible individuals. The enhanced subsidy under the ARP was set to expire in 2022, but the Inflation Reduction Act of 2022 (IRA) (4) extended it to 2025.

Table 1^2^ shows the expected premium contribution in coverage years 2020 (before the ARP) and 2021 (after the ARP).

**Table 1:** Expected premium contribution.

Expected premium contribution (coverage year 2020: before the ARP) (29)								
Annual household income (% of FPL)	Less than 133% FPL	133% FPL	138% FPL	150% FPL	200% FPL	250% FPL	300–400% FPL	400% FPL and above
Expected premium contribution (% of income)	2.06%	3.09%	3.39%	4.12%	6.49%	8.29%	9.78%	N/A
Expected premium contribution (coverage year 2021: after the ARP) (30)								
Annual household income (% of FPL)			Up to 150% FPL	200% FPL	250% FPL		300% FPL	400% FPL and above
Expected premium contribution (% of income)			0%	2%	4%		6%	8.5%

Above chart shows that before the ARP, subsidies were only available to those making less than 400% of the FPL. People with income four times or more the FPL fell into a “subsidy cliff” (meaning they made too much to qualify for subsidy) (5). After the ARP was passed in 2021, the subsidy cliff was removed and higher-income people (400% of FPL or more) became eligible for premium subsidy and their expected premium contribution is capped at 8.5% of their income. Additionally, existing eligible individuals now receive more generous subsidies, with people earning up to 150% of FPL now receiving health insurance for free when their expected premium contribution was 2.06–4.12% of their income before the enactment of the ARP. Those earning between 200 and 300% of the FPL also have less premium contribution with the ARP in place.

To demonstrate the premium subsidy calculation, let us consider a hypothetical 30-year-old woman with an annual income of $31,900 (250% of FPL of $12,760 for a single-person household in 2020^3^). Her annual premium contribution will be $2,644.51 (which is 8.29% of her income) in 2020. If the benchmark plan is priced at $6,000 for a 30-year-old single coverage, she would be eligible for a premium subsidy of $3,355.49 ($6,000 minus $2,644.51). Should the premium go up to $7,000, the premium subsidy would go up to $4,355.49 ($7,000 minus $2,644.51). Therefore, despite the premium increase, she would always pay the same premium contribution of $2,644.51. In 2021, her premium share is lowered to 4% under the ARP, so her premium contribution is $1,276 (=$3,1900*4%) and her premium subsidy is $4,724 (=$6,000–$1,276), an additional $1,368.51 (=$4,724–$3,355.49) from before the ARP.

Since only marketplace plans are eligible for premium subsidy, off-marketplace enrollment (when people purchase health insurance off state marketplaces) has been steadily declining. However, the rapid growth in marketplace enrollment in recent years (with a record breaking 21.4 million in 2024) has boosted the overall individual market with a 29% increase from 14.1 million in 2020 (the lowest since the ACA became law) to 18.2 million in early 2023 (6).

We observed an increased demand for gold plans (which had more generous coverage than silver plans) since 2021 when the enhanced premium subsidy came into existence. Our analysis of the insurer data also showed that insurers met the increased demand for gold plans. We also found that insurers offered more silver plans but less bronze plans than consumer demanded.

## Study data and methods

To examine the demand side of the individual market, we utilized two sources. The annual enrollment data by on- and off-marketplace were directly compiled from the Kaiser Family Foundation (KFF) without any manipulation (6). Annual enrollment data by metal types were also directly taken from the Centers for Medicare & Medicaid Services (CMS) enrollment reports without any further data work (7).

To examine the supply side of the individual market, we used the Robert Wood Johnson Foundation’s HIX Compare (8) (2014–2024), which contains comprehensive information on key characteristics of nearly every plan offered in the health insurance market, both on- and off-marketplace. The characteristics include plan market, metal tier, plan type^4^, premiums, deductibles, and maximum out of pocket expenses. Our study focused on the first two characteristics.

### Plan market

HIX Compare codes plan market in three exclusive categories, including on-marketplace-only plans, off-marketplace-only plans, and both-on-and-off-marketplace plans. We regrouped the three categories into two mutually exclusive categories: on-marketplace plans (which include on-marketplace-only plans and those offered both on- and off-marketplace) and off-marketplace-only plans (“off-marketplace plans” for short hereinafter).

### Metal tier

HIX Compare reports four metal types as mentioned in the introduction section: bronze, silver, gold, and platinum plans covering an average of 60, 70, 80, and 90% of the medical cost, respectively; as well as the catastrophic plan that is only available to people under 30 or people 30 or older with a hardship exemption or affordability exemption.

The HIX data are by year, state, carrier, and rating area. Each data observation indicates what health plan is offered by which carrier in which rating area of which state in what year, as well as that plan’s characteristics such as plan market and metal type. Only plans that were being sold were included in the annual datasets. There was no missing information on the key characteristics used in our study. We thus included all observations in our descriptive analysis without any sample selection.

There are several limitations with our data sources. HIX Compare did not indicate how discontinued plans or duplicate entries were handled. The two sources of enrollment data also did not discuss how mid-year entries/exits were reported.

## Study results

We conducted trend analysis of the enrollment data and the insurer data and reported our results below. We recognized that our study design was descriptive in nature and the results presented thus cannot establish causality.

### Number of insurers

The annual HIX Compare datasets track carriers’ plan offerings in each state’s designated rating areas. We sorted the data by year and carrier to examine the number of insurers each year. There was no sample selection involved as HIX Compare only included insurers that sold plans in at least one state at any given time during 2014–2024.

We tallied 244 insurers in the 2014–2024 HIX Compare datasets. The mean and median values of years that insurers stayed active during the study period were 6.71 and 8 years, respectively. Table 2 shows the number of insurers active for different lengths. For instance, 63 insurers (26%) were active during all 11 years (2014–2024), 32 insurers (13%) were active for 2 years, and 25 insurers (10%) were active for only one year. The entry and exit of insurers likely resulted in the market size fluctuation.

**Table 2:** Insurer years active.

# of insurers in HIX data	# of years active	% of insurers
63	11	26%
48	10	20%
8	9	3%
9	8	4%
5	7	2%
3	6	1%
4	5	2%
18	4	7%
29	3	12%
32	2	13%
25	1	10%

2014–2024 HIX compare individual market data.

Table 3 shows the nationwide number of active insurers and the median number of insurers on- and off-marketplace at the state level each year^5^. On- and off-marketplace enrollment data were also presented for comparison between demand and supply.

**Table 3:** Number of insurers vs. enrollment.

Year	Nationwide # of insurers	On-marketplace		Off-marketplace	
		Median # of insurers at state-level	National enrollment (in millions)	Median # of insurers at state-level	National enrollment (in millions)
2014	100	4	8	Not available	Not available
2015	184	5	11.7	6	9.6
2016	170	4	12.7	5	8.1
2017	164	3	12.2	4	6.4
2018	137	2	11.8	2	4.2
2019	132	3	11.4	2	3.6
2020	138	3	11.4	3	3.4
2021	146	5	12	3	3.6
2022	153	5	14.5	3	3.2
2023	159	5	16.4	3	2.5
2024	155	6	21.4	2	2.5

Data on numbers of insurers are derived from the 2014–2024 HIX Compare. Enrollment data (both on- and off-marketplace) are from KFF (https://www.kff.org/private-insurance/issue-brief/as-aca-marketplace-enrollment-reaches-record-high-fewer-are-buying-individual-market-coverage-elsewhere/). The 2025 off-marketplace enrollment of 2.5 million was KFF’s estimate. HIX Compare does not report any off-marketplace plans in 2014; hence there is no information on the median number of off-marketplace insurers for that year.

The initial marketplace enrollment went from 8 million in 2014 to 11.7 million in 2015 and 12.7 million in 2016. The market shrank in the next few years, with an enrollment of 12.2 million in 2017, 11.8 million in 2018, and 11.4 million in both 2019 and 2020. The past few years saw market expansion again, with 12 million enrollees in 2021, 14.5 million in 2022, 16.4 million in 2023, and 21.4 million in 2024. The rapid increase between 2021 and 2024 was likely due to the enhanced premium subsidy mentioned earlier. The off-marketplace enrollment was shown to be declining.

The number of insurers (both nationwide and at the state-level) was seen to largely track the movement of enrollment. When the marketplace expanded (or contracted), there were more (or fewer) insurers both at the national and state levels. The state level median number reached the highest of 6 in 2024, which likely contributed to the record-breaking enrollment.

There was also a strong association between the state-level median number of off-marketplace insurers and off-marketplace enrollment. We noted a largely downward trend, from a median number of 6 insurers at the state-level in 2015 to 2 in 2024. In the meantime, the off-marketplace enrollment went down from 9.6 million nationwide in 2015 to 2.5 million in 2024.

### Plan market distribution

**Figure 1:**
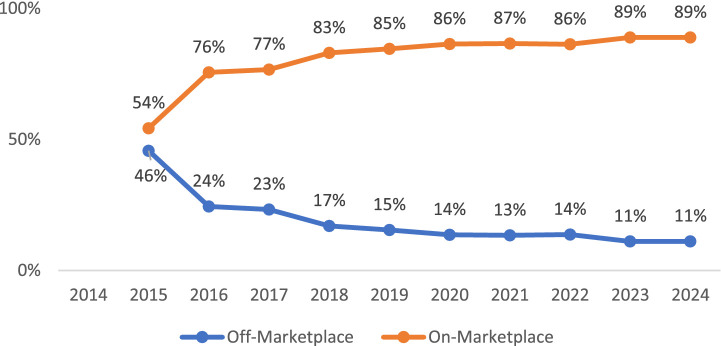
Plan market (national level). Author’s analysis of 2014–2024 HIX Compare. HIX compare does not report off-marketplace plans in 2014 so our results were for 2015–2024.

HIX Compare included only active health plans that were being sold and we included all of them in our tally^6^ of plans that were sold on or off marketplaces each year. The results were reported in Table 4 (counts) and Figure 1 (percentages). The percentage of on-marketplace plans went from 54% (=10,959/20186) in 2015 to an all-time high of 89% (=16,668/18744 = 14,916/16782) in 2023–2024. In the meantime, off-marketplace plan share declined from 46% (=9227/20186) in 2014 to an all-time low of 11% (=2076/18744 = 1866/16782) in 2023–2024.

**Table 4:** Plan market distribution.

Year	Total	On-marketplace	Off-marketplace
2015	20,186	10,959	9,227
2016	14,684	11,097	3,587
2017	12,375	9,490	2,885
2018	7,713	6,401	1,312
2019	8,926	7,547	1,379
2020	10,923	9,435	1,488
2021	13,234	11,460	1,774
2022	18,139	15,650	2,489
2023	18,744	16,668	2,076
2024	16,782	14,916	1,866

Author’s analysis of 2014–2024 HIX Compare. HIX Compare does not report off-marketplace plans in 2014 so our results were for 2015–2024.

### Metal tier distribution

**Figure 2:**
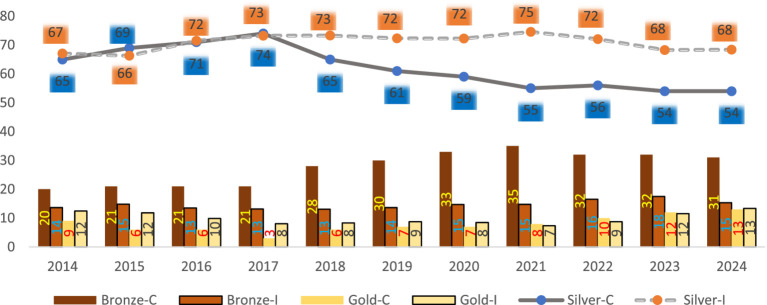
Marketplace plans: insurer supply (I) vs. consumer (C) demand (in %). Data on the metal plans offered by insurers (I) are calculated from the 2014–2024 HIX compare individual market datasets. Data on the metal plans purchased by consumers (C) are compiled from the CMS annual open enrollment reports. Platinum and catastrophic plans were not graphed as they accounted for a very small percentage.

Figure 2 compares the distribution of metal tiers supplied by insurers (I) vs. purchased by consumers (C) on marketplace. The consumer demand (C) was measured by the percentage of consumers purchasing various metal plans. This data was directly compiled from the CMS annual open enrollment reports without any manipulation.^7^

The CMS annual reports track plan enrollment data for every U. S. state (32 of which use the federal HealthCare.gov eligibility and enrollment platform and 19 run their own state-based Marketplaces). The total annual enrollment is defined as the count of consumers who selected a plan on marketplaces in a particular year. The percentage of consumers in each metal tier is the number of consumers that purchased that metal plan divided by the total enrollment. This percentage captures the consumer demand for different metal plans given the premium subsidy they may be eligible for. Without subsidies, consumers may choose a different plan based on their ability to pay the sticker price and other considerations. Since we aimed to examine how consumer demand evolved in the changing regulatory environment, we believe the CMS-reported percentages of consumers in various metal plans were a good measure to use for our study.

On the supply side, the ACA requires health plans sold on marketplaces to have specified levels of actuarial value in various metal tiers (with bronze plans covering an average of 60% of enrollees’ medical expenses, silver 70%, gold 80%, and platinum 90%). However, insurers are not mandated to offer all of the plans on marketplaces that they choose to enter. They may decide on a particular mix of health plans based on anticipated consumer demand, market competition, and other regulatory factors. We measured the insurer supply (I) by the percentages of metal plans offered on marketplaces in each year, which we calculated from HIX Compare by dividing the number of plans in each metal type by the total number of plans. Since HIX Compare only included active plans that were being sold (meaning every plan had pertinent information about plan details), our calculations included all the plans in the data without removing any observations.

We understand this supply measure was influenced by regulatory considerations. For instance, the ACA requires insurers to offer plans with cost-sharing reductions (CSR)^8^ to eligible individuals enrolled in silver plans. The CSR reduces out-of-pocket expenses for eligible enrollees without additional cost. It is considered an extra saving in additional to premium subsidy. Unlike premium subsidy, CSRs are only available in silver plans. This regulation may encourage eligible enrollees to seek more of silver plans and in turn motivate insurers to meet the demand. Our measure of market supply thus reflected insurer willingness to offer health plans given regulatory constraints and helped us understand the changing market supply over time in relation to consumer demand.

For both supply and demand, Figure 2 indicates that silver plans were the most popular^9^, followed by bronze and gold plans (platinum and catastrophic accounted for a much smaller percentages and were not shown on the graph). For instance, among all the plans purchased on marketplace in 2014, 65% were silver, 20% bronze, 9% gold, 5% platinum, and 2% catastrophic. The percent distribution for plans sold on marketplace in 2014 was 67% silver, 14% bronze, 12% gold, 4% platinum, and 2% catastrophic.

Figure 2 shows a disconnect between supply of and demand for silver and bronze plans. Between 2014 and 2017, the demand for and the supply of silver plans were comparable. For instance, in 2017, 73% of marketplace plans sold were silver plans, while 74% of plans purchased on marketplace were silver. However, from 2018 to 2024, insurers were offering a higher percentage of silver plans than consumers demanded. For instance, in 2021, 75% of plans offered on marketplace were silver plans, but only 55% of plans purchased on marketplace were silver. In the meantime, insurers were offering fewer bronze plans than consumers demanded. For instance, in 2021, 15% of plans offered by insurers on marketplace were bronze, but 35% of plans purchased on marketplace were bronze.

From 2014 to 2020, consumers were demanding fewer gold plans than offered. But starting in 2021, consumers were demanding more gold plans than offered. In 2021, 7% of plans offered were gold but 8% purchased were gold. In 2023 and 2024, insurers were catching up and offering about the same percentage of gold plans as demanded.

We conducted separate chi-square tests for each of the three major metal plans to see whether the insurer supply matches the consumer demand.^10^ Table 5 shows the test results. The *p*-values were smaller than 0.05 for silver and bronze plans. Therefore, we reject the null hypotheses at the 0.05 significance level that insurer offerings of silver and bronze plans are the same as those demanded by consumers. In other words, there is a statistically significant mismatch between supply of and demand for silver and bronze plans. On the other hand, gold plan chi-square test had a p-value of 0.435 and failed to reject the hypothesis that this metal type’s supply and demand are the same. This is consistent with our observation that insurers offered about the same percentage of gold plans as demanded in 2023 and 2024.

**Table 5:** Chi-square tests of consumer demand (C) (%) vs. insurer supply (I) (%).

Year	Observed	Expected	Observed	Expected	Observed	Expected
Silver-C	Silver-I	Bronze-C	Bronze-I	Gold-C	Gold-I
2014	65	67	20	14	9	12
2015	69	66	21	15	6	12
2016	71	72	21	13	6	10
2017	74	73	21	13	3	8
2018	65	73	28	13	6	8
2019	61	72	30	14	7	9
2020	59	72	33	15	7	8
2021	55	75	35	15	8	7
2022	56	72	32	16	10	9
2023	54	68	32	18	12	12
2024	54	68	31	15	13	13
Chi-square statistic	20.09		143.41		10.06	
*p*-value	0.028		<0.0001		0.435	

Data on the metal plans offered by insurers (I) are calculated from the 2014–2024 HIX Compare Individual Market datasets. Data on the metal plans purchased by consumers (C) are compiled from the CMS annual open enrollment reports.

Figure 2 also shows that as the metal tier distributions changed for both supply and demand, a trend emerged since 2021—there were lower percentages of silver plans sold and purchased (reaching an all-time low of 54% on the demand side and second-lowest percentage of 68% on the supply side), but more gold plans sold and purchased, reaching an all-time high of 13% for both demand and supply.^11^ This observation is likely due to consumers’ trading up with the enhanced premium subsidy.

## Discussion and policy implication

As the government took actions to enhance premium subsidy, it’d be important for health insurers to work in sync with the government to further improve consumer access to better coverage at reasonable cost. It’d be counter-productive if health insurers’ coverage design offset the benefit that consumers gained from the enhanced premium subsidy.

Our study showed that the number of insurers moved in sync with the fluctuation in total enrollment. Further analysis of various metal types indicated a disconnect between demand and supply, with insurers offering more silver but less bronze plans than consumers demanded. But insurers did offer more gold plans as consumers traded up for better coverage (gold plans had lower out of pocket expenses than silver plans) since 2021. While our descriptive study does not establish causality between enhanced premium subsidy and consumers choice, the observed pattern between the two is consistent with the health plan choice literature.

A study on consumer choice among health insurance options (9) noted that most people are drawn to plans with the lowest premiums and out-of-pocket expenses when other factors are comparable. Given the difficulty of predicting healthcare needs and out-of-pocket expenses, the known premium cost is considered most salient and a main determinant of consumer choice for many consumers. An examination of the National Medical Care Expenditure Survey indicated that price was a significant determinant of employee choice of health plans (10). A five-state analysis of marketplace plans in 2015 reported that consumers in California, Connecticut, Maryland, New York, and Rhode Island made their plan choices mainly based on price (11). A two-state study in 2023 also found evidence that price was the main factor that drove consumer choice of marketplace plans in California and New York (12).

Several other studies estimated price elasticities. A study of 1,553 subscribers in three Minneapolis-St Paul HMOs (Health Maintenance Organizations) found that a $5.00 increase in monthly premium would lead to a two-thirds increase in that HMO’s disenrollment rate (13). When the University of California (UC) implemented a policy change that resulted in an increase in employee-share premium for about a third of its workforce, employees facing premium increases of less than $10 were found to be roughly five times as likely to switch to lower-cost plans as those whose premiums remained constant (14). A panel data study of Stanford University group insurance benefits also found large price elasticities ranging from −3.7 to −6.2 in one scenario (15).

The enhanced premium subsidy reduces net premiums for consumers. On average, the ARP saved an estimated $705 per year in premium payment for all subsidized enrollees (16). Results from our descriptive analysis showed steady increases in overall marketplace enrollment and heightened demand for gold plans post-ARP.

The enhanced subsidy is set to expire on December 31, 2025. If not renewed, consumers are expected to face over 75% of premium increase (16). Those in states that have not expanded Medicaid^12^ may experience even higher premiums as studies have shown that premiums of marketplace plans in non-expansion states were 11% higher than those in expansion-states (17). Given consumers’ price sensitivity as documented in literature, some may choose to go uninsured should the enhanced premium subsidy expire, especially when there is no longer an individual mandate for purchasing health insurance (18). A study predicted a marketplace enrollment drop from 22.8 million in 2025 to 18.9 million in 2026 and fall to 15.4 million in 2030 (16).

People making 400% of more of the FPL will no longer be eligible for the enhanced premium subsidy should it expire. They may pursue short-term, limited duration insurance (STLDI) off marketplaces. STLDI is exempted from the ACA regulations of offering essential benefits and meeting other requirements. While less expensive, STLDI lacks the same protection for consumers as ACA-compliant plans (19).

Those still eligible for regular premium subsidy may trade down on marketplace plans. Some may qualify for Medicaid, but if they live in states that have Medicaid waivers^13^ restricting eligibility^14^ (20), they may struggle to get the coverage they need.

Above are some examples of potential consequences that consumers may face with the expiration of the enhanced premium subsidy. While the One Big Beautiful Bill Act (OBBBA) (21) signed into law on July 4, 2025 did not extend the enhanced premium subsidy (22), the U. S. Congress may still consider taking actions before the year end to avoid the expiration of subsidy enhancement; otherwise, people with incomes 400% of poverty will fall into the subsidy cliff again and no longer qualify for any premium subsidy. Lower-income enrollees who are still eligible but will receive less subsidy may “trade down” and revert back to less generous plans, which will likely influence insurers’ plan offering. The HIX Compare data, once updated in 2025 and beyond, can be used to reexamine insurer strategy in anticipation of or in response to consumer demand change should the enhanced premium subsidy indeed be allowed to expire on December 31, 2025.

## Limitations and future research

Our research was designed to be descriptive and did not test whether the enhanced premium subsidy caused consumers to change their insurance purchase behavior. Previous studies showed that in addition to price, many other factors also influence consumer choice in health insurance purchase, such as quality of physicians (23), quality of health plans (24), number of plans available (25, 26), health insurance literacy and comprehension (27, 28). Additionally, consumer choice may be affected by market dynamics as insurers may use different pricing strategies in different states and under different circumstances (such as during the pandemic period, which preceded the enactment of the ARP) in the ever-changing regulatory environment. To fully explore the casualty between the enhanced premium subsidy and consumer choice, future research may extend our observational study and consider including these confounding factors in the analysis.

## Data Availability

The raw data supporting the conclusions of this article will be made available by the authors, without undue reservation.

## Footnotes

^1^The percentage of household income varies with household income and also changes from year to year.

^2^Compiled from https://www.healthreformbeyondthebasics.org/reference-guide-yearly-thresholds/

^3^ https://aspe.hhs.gov/topics/poverty-economic-mobility/poverty-guidelines/prior-hhs-poverty-guidelines-federal-register-references/2020-poverty-guidelines

^4^Five plan types are reported: PPO (preferred providers’ organization), HMO (health maintenance organization), POS (point of service), EPO (exclusive provider organization) or other.

^5^We sorted the data by year, state, and carrier to tally the number of insurers in each state each year. We then calculated the median numbers of insurers each year.

^6^We sorted the data by year and plan ID before doing the counts and percentages.

^7^The annual open enrollment reports include the percentages of consumers purchasing different metal types on marketplaces. For instance, such percentages were presented in Table 5 (pages 14–15) of the 2024 report https://www.cms.gov/files/document/health-insurance-exchanges-2024-open-enrollment-report-final.pdf.

^8^ https://www.healthcare.gov/lower-costs/save-on-out-of-pocket-costs/

^9^Likely in part due to the CSR mentioned above. According to CMS (https://www.cms.gov/files/document/health-insurance-exchanges-2024-open-enrollment-report-final.pdf), 50% of enrollees received CSRs in 2024.

^10^The null hypothesis is that there is no difference between the expected percentage of each metal plan supplied by insurers and the observed percentage of the metal plan expected by consumers. The alternate hypothesis is that there is a difference between the two.

^11^We did a one-tailed t-test and found that consumer purchased a significantly higher percentage of gold plans post-ARP (2021–2024) than pre-ARP period (2014–2020) at the 0.01 significance level. The demand for silver plans was shown to be significantly lower post-ARP than pre-ARP (also at the 0.01 significance level).

^12^According to Kaiser Family Foundation (https://www.kff.org/medicaid/status-of-state-medicaid-expansion-decisions/), 41 states (including Washington DC) have expanded Medicaid coverage (a joint federal and state program that covers healthcare costs for lower income people) to nearly all adults with incomes up to 138% of the FPL.

^13^According to Kaiser Family Foundation (https://www.kff.org/medicaid/medicaid-waiver-tracker-approved-and-pending-section-1115-waivers-by-state/), nearly every state has Medicaid waivers, which allow states to test new approaches to offer Medicaid than required by federal statute.

^14^While most waivers aim to broaden coverage, some states have waivers that restrict eligibility (such as charging enrollees premiums or locking out enrollees disenrolled for unpaid premium) (https://www.kff.org/medicaid/medicaid-waiver-priorities-under-the-trump-and-biden-harris-administrations/).
